# The effectiveness of a web-based intervention for Japanese adults with problem drinking: An online randomized controlled trial

**DOI:** 10.1016/j.abrep.2021.100400

**Published:** 2021-12-14

**Authors:** Toshitaka Hamamura, Shinichiro Suganuma, Ayumi Takano, Toshihiko Matsumoto, Haruhiko Shimoyama

**Affiliations:** a National Center for Cognitive Behavior Therapy and Research, National Center of Neurology and Psychiatry, Tokyo, Japan; b Japan Society for the Promotion of Science, Tokyo, Japan; c Department of Humanities, National Defense Academy, Kanagawa, Japan; d Department of Mental Health and Psychiatric Nursing, Tokyo Medical and Dental University, Tokyo, Japan; e Department of Drug Dependence Research, National Center of Neurology and Psychiatry, Tokyo, Japan; f Department of Integrated Educational Sciences, University of Tokyo, Tokyo, Japan

**Keywords:** Alcohol outcome expectancy, Personalized normative feedback, Problem drinking, Randomized controlled trial, Web-based intervention

## Abstract

**Aims:**

This study aimed (1) to delineate how a web-based intervention affects the problem drinking behaviors of Japanese adults and (2) to examine the moderating effects of disorder levels and alcohol outcome expectancies on intervention outcomes.

**Methods:**

We implemented an online two-armed parallel-group randomized controlled trial with 546 Japanese adults. Adults aged 20 years or older and who scored eight or higher on the Alcohol Use Disorder Identification Test were included in this study. Participants were randomly allocated to the intervention group or the waitlist/control group. The intervention comprised assessment of drinking behavior, personalized normative feedback, psychoeducation about the consequences of problem drinking, and a short quiz. The outcomes were weekly drinking quantity and abstinent days, largest drinking quantity in one day, and alcohol-related consequences reported at baseline and at one-, two-, and six-month follow-ups. A mixed-effects model regression was conducted to compare the intervention and control groups.

**Results:**

The attrition rates at each follow-up were 52.93%, 49.45%, and 32.60%, respectively. The time × condition interaction effect on weekly drinking quantity was significant at the two- and six-month follow-ups, *d* = 0.28, 95% CI [0.04, 0.51], *d* = 0.34, 95% CI [0.05, 0.63], respectively. Moderations related to the intervention effect were not statistically significant.

**Conclusion:**

A web-based intervention was found to be effective for two and six months only on drinking quantity measures of Japanese adults with problem drinking. Limitations including high drop-out rates in are discussed.

## Highlights

• We conducted an online randomized controlled trial for a web-based intervention.
• The intervention comprised normative comparison, psychoeducation, and a short quiz.
• Participants were Japanese adults aged 20 years or older and scored AUDIT ≥ 8.
• The weekly drinking quantity at the two- and six-month follow-ups decreased.

## 1 Introduction

The literature suggests that individuals with problem drinking are often reluctant to seek professional help (Cunningham & Breslin, 2004, Mekonen et al., 2021). One way to overcome this problem is to utilize computerized interventions from which people receive interventions through technology instead of a face-to-face procedure. In addition to being more accessible to more individuals via the Internet, the use of computerized interventions can be less expensive (Elliott, Carey, & Bolles, 2008) and reduce the stigma associated with alcohol use disorder (AUD) treatment as some individuals prefer to receive treatment anonymously (Lapham et al., 2012).

Previous studies have demonstrated the efficacy of computerized interventions that diminish behaviors related to problem drinking, although the effect sizes are small, and interventions last for six months or less (Khadjesari, Murray, Hewitt, Hartley, & Godfrey, 2010, Riper et al., 2018). When compared to face-to-face interventions, computerized interventions have shown similar effects both in general adult population and mandated college students (Carey, Scott-Sheldon, Garey, & Elliott, 2016, Kaner et al., 2017). The literature suggests the effectiveness of personalized normative feedback (PNF), a type of intervention that informs individuals about their behaviors that deviate from the reported norm. The efficacy of computerized interventions with PNF for problem drinking has been demonstrated among adults in the general adult population in Canada (Cunningham, Wild, Cordingley, Van Mierlo, & Humphreys, 2009) and in particular populations in the U.S., including young war veterans (Pedersen, Parast, Marshall, Schell, & Neighbors, 2017) emerging adults with risky drinking (Lau-Barraco, Braitman, & Stamates, 2018), and young adults with alcohol-related risk-taking behaviors (Lewis et al., 2019). Interventions with PNF often include other components such as psychoeducation, harm reduction strategies, calories from alcohol, and motivations for change to help individuals find ways to drink moderately (Cunningham, Wild, Cordingley, Van Mierlo, & Humphreys, 2009, Riper et al., 2009).

In Japan, a small portion of individuals with alcohol-related problems seek professional help—for example, 5.4% in 2005 (Cho, 2011). Meanwhile, a study using a national sample suggested that 24.6% of men and 3.2% of women drink at least at an at-risk level, scoring ≥ 8 on the AUD Identification Test (AUDIT; Osaki, 2014). Despite this, few studies have examined the effects of computerized interventions on problem drinking among Japanese. Among male employees, discussing the risks and appropriate behaviors of alcohol consumption via email did not decrease alcohol consumption (Araki, Hashimoto, Kono, Matsuki, & Yano, 2006). College students receiving an ethanol patch test, which provides information related to genetic background associated with alcohol tolerance, and watching a video about alcohol-related health risks in a classroom setting, increased their knowledge about alcohol-related problems (Geshi, Hirokawa, Taniguchi, Fujii, & Kawakami, 2007). However, the number of actual alcohol-related problems did not decrease at two-month follow-up (Geshi et al., 2007).

Previous studies have examined moderating factors for computerized interventions. Meta-analyses have demonstrated that computerized interventions reduced heavy drinking frequency with a medium effect size among university students selected as heavy drinkers (Carey, Scott-Sheldon, Elliott, Garey, & Carey, 2012). Similarly, computerized interventions were more effective among those who drank more than 21 standard units of alcohol weekly (Riper et al., 2018). Canadian adults scoring ≥ 11 on the AUDIT reported greater reduction of drinking quantity (six drinks per week reduction) at the six-month follow-up while the effect was not found among those scoring ≤ 10 on the AUDIT after receiving an intervention with PNF and psychoeducation (Cunningham et al., 2009). However, among German at-risk drinkers, the effect of computerized feedback about their alcohol use on drinking quantity was greater among those scoring ≤ 8 on the AUDIT (Baumann et al., 2018).

Alcohol outcome (AO) expectancies are associated with problem drinking, but little is known about whether they moderate the relationship between computerized interventions and problem drinking. AO expectancies refer to one’s beliefs about the effects of alcohol on one’s psychological and physiological states (Jones, Corbin, & Fromme, 2001). Positive AO expectancies refer to beliefs about positive effects of alcohol on one’s psychological and physiological states as alcohol consumption enhances the pleasantness of experiences or reduces feelings of tension (Brown, Goldman, Inn, & Anderson, 1980). Negative AO expectancies are beliefs about deleterious consequences of drinking and generally motivate people to drink less (Jones et al., 2001). A reduction of positive AO expectancies was associated with a reduction of problem drinking such as drinking quantity while reduction of negative AO expectancies was associated with more frequent drinking (Goldman, Del Boca, & Darkes, 1999, McMahon, Jones, & O’Donnell, 1994, Monk & Heim, 2013). Expectancy challenges, interventions aimed at changing people’s beliefs regarding drinking, can be a tool to help lower the incidence of problem drinking (Carey, Scott-Sheldon, Elliott, Garey, & Carey, 2012, Goldman, Del Boca, & Darkes, 1999, Scott-Sheldon, Terry, Carey, Garey, & Carey, 2012). If positive AO expectancies are associated with problem drinking and decrease with problem drinking through an intervention, AO expectancies may function as moderators of computerized interventions on problem drinking as components such as PNF often challenge negative consequences of problem drinking.

Recent web-based computerized interventions that include PNF have the potential to reduce problem drinking despite the reported small effect sizes and limited duration. However, this evidence has not been generalized to Japanese adults. Meanwhile, levels of AUD appear to moderate the effects of computerized interventions on problem drinking (Cunningham et al., 2009), but this moderation among adults needs further investigation. Additionally, no studies have examined AO expectancies as moderators of computerized interventions when they are related to problem drinking. We believe that the novelty of the research lies in examining the efficacy of computerized interventions among Japanese adults and the moderating effects of AO on the intervention. Specifically, having lower positive AO expectancies, beliefs that alcohol consumption serves as a mood-regulating or stress-coping strategy, likely leads to a decrease in problem drinking. On the other hand, having higher negative AO expectancies, beliefs that alcohol consumption brings about negative consequences, is also likely to lead to a decrease in problem drinking.

Based on the aforementioned descriptions, we examined whether we could reject the following primary null hypothesis: Japanese adults who receive a web-based intervention with PNF do not report reduced weekly drinking quantity at the six-month follow-up compared to those on the waitlist. As exploratory analyses, we examined whether the same results as the primary hypothesis would be obtained for weekly abstinent days, largest drinking quantity in one day, and alcohol-related consequences. Additionally, we examined whether or not participant s’ lower level of baseline AUD and positive AO expectancies would lower the effects of the intervention for reducing problem drinking while stronger negative AO expectancies would strengthen the effects of the intervention.

## 2 Methods

### 2.1 Participants and inclusion criteria

Japanese adults with problem drinking were recruited via Japanese crowdsourcing websites (Crowdworks, 2021, Lancers, 2021). The inclusion criteria were being at least 20 years old, the legal age for drinking in Japan, and a score of ≥8 on the AUDIT.

### 2.2 Trial design and procedure

We conducted a two-armed parallel-group randomized controlled trial via the Internet; thus, all participation occurred on a web browser. First, potential participants saw the online post on the crowdsourcing websites regarding recruitment and were directed to the intervention webpage, which first assessed if they met the inclusion criteria. Those who met the criteria were allowed to read the informed consent form, while those who did not were informed about their ineligibility. After understanding the nature of the trial and agreeing to participate, all participants were asked to provide their consent and completed the baseline measures.

The website created for this trial automatically allocated participants to either the intervention or the waitlist using computer-generated numbers with a ratio of 1 to 1. After the completion of the baseline measures, the intervention group received the intervention immediately while participants in the waitlist group were notified that they would receive the intervention after the six-month follow-up. Therefore, participants were blinded to their condition until they completed the baseline measures. The investigators did not have prior knowledge about participants’ allocation until data analysis.

Participants were asked to complete the outcome measures at four points: at baseline and at one-, two-, and six-month follow-ups. At each evaluation, participants were asked to enter self-assessed information about their drinking patterns since their last participation. If participants missed a follow-up, they were still invited to complete the subsequent follow-up measures. At the end of the trial, participants were debriefed about the nature of the study. After completion of all follow-ups, participants received ¥1200 in remuneration through the recruitment websites: ¥800 at two-month follow-up and ¥400 at six-month follow-up (roughly US $10 in total on June 1, 2018). This study did not compensate participants at baseline and one-month follow-up to prevent early dropouts.

### 2.3 Intervention

The intervention was developed to be accessible through a web. The intervention comprised four components: (1) assessment of drinking, (2) PNF, (3) psychoeducation, and (4) a short quiz. The assessment, PNF, and psychoeducation parts were developed based on an intervention that demonstrated efficacy in a previous study (i.e., Cunningham et al., 2009). The assessment component asked participants about their drinking quantity and frequency, largest drinking quantity in a day, negative consequences related to drinking, and average money spent in a day of drinking. They also completed the AUDIT and provided demographic information. The PNF component provided feedback based on the entries from the assessment component, such as their typical drinking quantity, frequency of risky drinking, and level of AUD, and their estimated drinking quantity, financial costs, and caloric intake in one year. Each participant’s reported drinking quantity was compared with the averages of others of the same sex and age range. The psychoeducation component provided information about the negative impact of problematic drinking on psychological and physiological functioning and ways to reduce these negative consequences. Finally, the quiz component provided three multiple-choice questions from the psychoeducation component. The quiz was not part of the original study (Cunningham et al., 2009) but was included to help participants understand the materials. Participants were required to provide the correct answer to all three questions. The intervention took about 20 min to complete.

### 2.4 Measures

**Alcohol outcome (AO) expectancies.** The Japanese version of the AO Expectancies Scale (Leigh & Stacy, 1993, Sakurai, 1997) was used to measure positive and negative AO expectancies. This questionnaire starts with a statement, “In what state of mind do you feel yourself in when drinking alcohol?” Responses are made using a 6-point Likert scale (1 = *no chance*, 6 = *certain to happen*). This study used 20 of the original 51 items to measure four factors. We categorized mood enhancement and stress coping as positive AO expectancies and physical ailments and dysphoria as negative AO expectancies.

**Alcohol use disorder**. The Japanese version of the AUDIT (Babor, John, John, & Monteiro, 2011) was used to measure participants’ alcohol-related problems. It comprises 10 items with responses on a 5-point scale; response options differ for each item. The first three items measure drinking quantity and frequency, the next four measure dependence symptoms, and the last four measure harmful alcohol use.

**Weekly drinking quantity.** The Daily Drinking Questionnaire (Collins, Parks, & Marlatt, 1985) was used to measure participants’ drinking quantity each day. Participants were asked to recall their typical weekly behaviors and tasks and to indicate how many drinks they would consume each day—from Monday to Sunday. A weekly drinking score was calculated by summing their drinking reports for all seven days.

**Weekly abstinent days**. Using the weekly drinking quantity, we coded “1” for each day in which participants reported having drunk at least 1 unit of alcohol. The score was seven minus the sum of each day that was coded 1.

**Largest drinking quantity in one day.** Participants were asked, “When you drank the most in a day, how many drinks did you have?” They were asked the same question for “during the past 12 months” at baseline and “during the previous month” for the follow-ups.

**Alcohol-related consequences**. Part of the intervention includes measuring and revealing areas of one’s life that have been affected by drinking, including interpersonal relationships, health, satisfaction with life, marital relationship, employment, and finances. Participants answered whether their drinking had affected these areas of life by responding “yes” or “no” in the past 12 months at baseline and during the past month at each follow-up. The scale score is the sum of the items with the “yes” response and can range from 0 to 6. The items used were from the original intervention (Cunningham et al., 2009).

The predetermined primary outcome was the weekly drinking quantity. The secondary outcomes were weekly abstinent days, largest drinking quantity in one day, and alcohol-related consequences. Demographic information and the AO expectancies measure were completed only at baseline.

### 2.5 Ethical considerations

The research ethics board at the University of Tokyo approved this study (No. 17-174). All procedures followed the UMIN Clinical Trial Registry (R000034388), and details of the trial can be found in a published protocol (Hamamura, Suganuma, Takano, Matsumoto, & Shimoyama, 2018). The possible harm of this intervention relates to its inadequacy for those with severe AUD. If participants scored 20 or higher on the AUDIT during the intervention, the PNF portion of the intervention was configured to recommend they seek professional help. There was no reporting of any referral during the trial.

### 2.6 Statistical analyses

A logarithmic transformation was applied on variables with a positively skewed distribution. We used a mixed-effects model regression to examine the condition × time interaction effects on the primary and secondary outcomes. We treated these interactions as fixed effects, and subjects, intercepts, and slopes as random effects. For effect size, Cohen’s *d* was calculated by the following formula suggested by Feingold (2013):

$$d=\frac{b}{\mathrm{SD}}$$

where *b* denotes an unstandardized regression coefficient of the condition × time interaction effect at a specific follow-up. For the moderation effects of AUDIT and positive and negative AO expectancies, we added each moderator into the model separately and compared the model including the three-way interaction effect with the model excluding the three-way interaction. All analyses were performed using R 4.1.0 (R Core Team, 2021).

### 2.7 Power analysis

A power analysis revealed that the minimal sample size was 787 (393.4 participants in each group). We considered a statistical power of 80% and a significance level set at *p* < .05 for a small effect (Cohen’s *d* = 0.2) of the intervention on the weekly drinking quantity at the six-month follow-up from a previous meta-analysis (Dedert et al., 2015). A previous randomized controlled trial with a similar intervention estimated 170 participants, using a mixed-effects model (Cunningham, Godinho, & Kushnir, 2017). Among individuals with problem drinking, the drop-out ratio was 55% (Postel et al., 2011). Based on these studies, we aimed to recruit 600 participants as reported in the published protocol (Hamamura et al., 2018).

## 3 Results

### 3.1 Attrition and participant characteristics

**Fig. 1:**
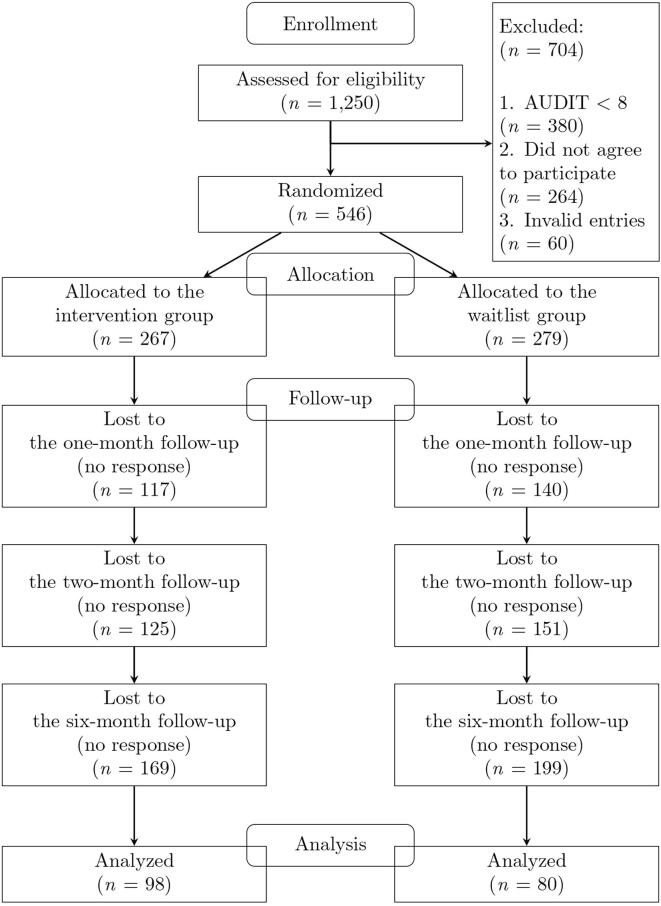
CONSORT flowchart of the trial.

Fig. 1 shows a flow chart of this study from CONSORT (Consolidated Standards of Reporting Trials; Schulz, Altman, & Moher, 2010). Recruitment, baseline measurement, and the intervention took place between January and February 2018. In total, 1250 Japanese individuals were screened for inclusion in the study. Among these, 546 (43.68%) met the criteria. Among the final participants, 236 (43.22%) were women, and the mean age was 36.66 (*SD* = 10.50). Based on random assignment, the intervention group had 267 participants, and the waitlist group had 279 participants. At the one-month follow-up, 289 participants (52.93%) responded to the questions: 150 (56.18%) in the intervention and 139 (49.82%) in the waitlist group. At the two-month follow-up, 270 participants (49.45%) responded: 142 (53.18%) in the intervention and 128 (45.88%) in the waitlist group. At the six-month follow-up, which was held between July and August 2018, 178 participants (32.60%) responded: 98 (36.70%) in the intervention and 80 (28.67%) in the waitlist group. Due to the high attrition, we did not impute missing data. Since a mixed-effects model regression analysis can be performed with missing data, subsequent analyses included missing data. Multiple analysis of variance showed significant demographic differences between participants with missing responses and participants without missing responses, *F*(1, 544) = 8.58, *p* < .001, Wilks’ λ = 0.97. Participants with missing responses were younger than those without missing responses, *F*(1, 544) = 16.95, *p* < .001, η^2^ = 0.03. The sex difference was not statistically significant, *F*(1, 544) = 0.54, *p* = .46, η^2^ = 0.001. No attrition due to adverse events was reported.

### 3.2 Effects of the intervention

**Fig. 2:**
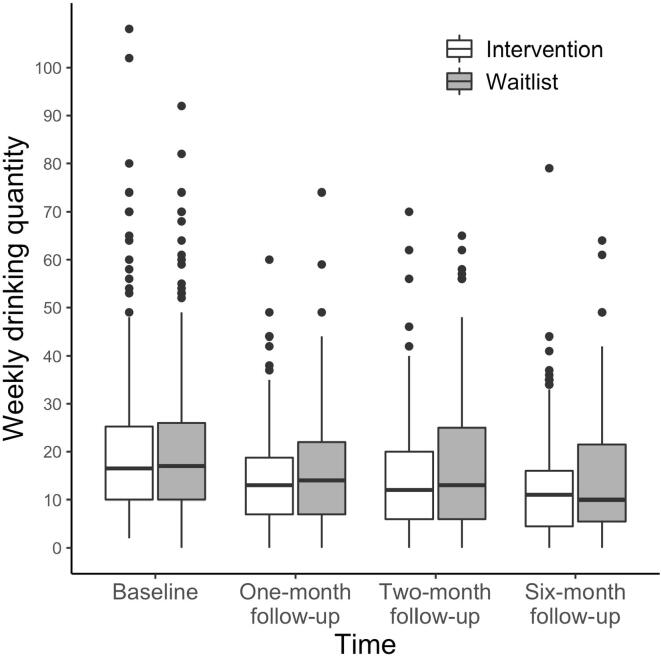
Participants’ weekly drinking quantity by condition and time.

Table 1 shows the mean, standard deviation, median, and reliability (ω) of the scales. Logarithmic transformation was applied to all four outcome measures due to their positive skewness. The condition × time interaction effects on weekly drinking quantity at the six-month follow-up were statistically significant, supporting our primary hypothesis, *d* = 0.34, 95% CI [0.05, 0.63]. The interaction effect was also statistically significant at the two-month follow-up, *d* = 0.28, 95% CI [0.04, 0.51] (Fig. 2). We calculated a mean reduction from baseline to each point and compared them between the intervention and waitlist group: the between-group differences were 2.87 and 1.94 fewer drinks at the two- and six-month follow-up, respectively. Fig. 2 shows participants’ average weekly drinking quantity by condition and time. None of the condition × time interaction effects on weekly abstinent days, largest drinking quantity in one day, and alcohol-related consequences were statistically significant. Table 2 shows the results of fixed effects.

**Table 1:** Mean, standard deviation, median, and reliability (ω) of each variable by condition and time.

Measures	Time	Intervention			Waitlist			ω
		Mean	*SD*	Median	Mean	*SD*	Median	
AUDIT	Baseline	13.89	5.22	12	14.14	5.35	13	0.67
Positive AOE	Baseline	42.18	7.14	43	41.47	7.49	42	0.84
Negative AOE	Baseline	23.29	7.08	23	24.04	6.70	23	0.77
Weekly drinking quantity	Baseline	21.06	16.14	16.5	20.78	15.26	17	0.89
	One-month FU	14.51	10.64	13	16.37	12.54	14	0.89
	Two-month FU	14.77	12.54	12	17.37	14.95	13	0.89
	Six-month FU	13.65	12.57	11	15.70	14.19	10	0.93
Weekly abstinent days	Baseline	1.55	1.89	0	1.50	1.81	1	0.81
	One-month FU	2.21	2.17	2	1.97	2.11	1	0.84
	Two-month FU	2.46	2.30	2	2.19	2.26	2	0.86
	Six-month FU	2.50	2.38	2	1.96	2.21	1	0.87
Largest drinking quantity in one day	Baseline	9.77	5.63	8	10.34	5.73	10	–
	One-month FU	6.48	4.02	5.5	6.78	3.79	6	–
	Two-month FU	5.90	3.79	5	6.53	3.88	6	–
	Six-month FU	5.41	4.47	4	6.46	5.43	5.5	–
Alcohol-related consequences	Baseline	2.02	1.76	2	1.88	1.71	1	0.71
	One-month FU	1.09	1.45	1	0.93	1.31	0	0.72
	Two-month FU	1.06	1.59	0	0.88	1.31	0	0.76
	Six-month FU	1.10	1.55	0	0.90	1.37	0	0.77

*Note*. AUDIT = Alcohol use disorder identification test. AOE = alcohol outcome expectancies. FU = Follow-up. SD = standard deviation.

**Table 2:** Parameter estimates on the outcome variables.

Outcome and fixed effect	*b*	*SE*	*t*	*p*	*d*	95% CI
Weekly drinking quantity						
Intercept	2.87	0.04	68.99	>0.000	4.24	4.12, 4.36
Condition	−0.001	0.06	−0.03	0.980	−0.002	−0.17, 0.17
Time: One-month FU	−0.35	0.05	−7.77	>0.000	−0.52	−0.65, −0.39
Time: Two-month FU	−0.47	0.06	−8.19	>0.000	−0.69	−0.85, −0.52
Time: Six-month FU	−0.62	0.07	−9.23	>0.000	−0.92	−1.11, −0.72
Condition × Time: One-month FU	0.08	0.07	1.28	0.202	0.12	−0.07, 0.31
Condition × Time: Two-month FU	0.19	0.08	2.25	0.025	0.28	0.04, 0.51
Condition × Time: Six-month FU	0.23	0.10	2.34	0.020	0.34	0.05, 0.63
Weekly abstinent days						
Intercept	0.67	0.04	15.26	>0.000	0.94	0.82, 1.06
Condition	−0.02	0.06	−0.24	0.81	−0.02	−0.19, 0.15
Time: One-month FU	0.20	0.04	5.01	>0.000	0.28	0.17, 0.40
Time: Two-month FU	0.31	0.05	6.47	>0.000	0.43	0.3, 0.56
Time: Six-month FU	0.34	0.06	5.45	>0.000	0.47	0.3, 0.64
Condition × Time: One-month FU	−0.03	0.06	−0.55	0.58	−0.05	−0.21, 0.12
Condition × Time: Two-month FU	−0.10	0.07	−1.49	0.14	−0.14	−0.33, 0.05
Condition × Time: Six-month FU	−0.16	0.09	−1.70	0.09	−0.22	−0.47, 0.03
Largest drinking quantity in one day						
Intercept	2.26	0.03	76.81	>0.000	4.72	4.60, 4.84
Condition	0.05	0.04	1.24	0.22	0.10	−0.06, 0.28
Time: One-month FU	−0.37	0.042	−8.88	>0.000	−0.78	−0.96, −0.61
Time: Two-month FU	−0.48	0.04	−10.91	>0.000	−1.00	−1.18, −0.82
Time: Six-month FU	−0.60	0.06	−10.46	>0.000	−1.24	−1.48, −1.01
Condition × Time: One-month FU	0.01	0.06	0.12	0.90	0.02	−0.23, −0.26
Condition × Time: Two-month FU	0.06	0.06	0.92	0.36	0.12	−0.14, 0.38
Condition × Time: Six-month FU	0.14	0.08	1.66	0.10	0.29	−0.05, 0.64
Alcohol-related consequences						
Intercept	0.92	0.04	23.90	>0.000	1.47	1.35, 1.59
Condition	−0.05	0.05	−0.94	0.35	−0.08	−0.25, 0.09
Time: One-month FU	−0.34	0.05	−7.17	>0.000	−0.55	−0.69, −0.40
Time: Two-month FU	−0.40	0.05	−7.85	>0.000	−0.64	−0.80, −0.48
Time: Six-month FU	−0.37	0.05	−6.90	>0.000	−0.59	−0.75, −0.42
Condition × Time: One-month FU	−0.04	0.07	−0.61	0.54	−0.07	−0.28, 0.15
Condition × Time: Two-month FU	−0.003	0.07	−0.04	0.97	−0.005	−0.23, 0.23
Condition × Time: Six-month FU	−0.02	0.08	−0.31	0.76	−0.04	−0.29, 0.21

*Note.* CI = Confidence interval. FU = Follow-up. SE = Standard error.

### 3.3 Moderation analyses

Only the model with positive AO expectancies on alcohol-related consequences improved, χ^2^(27) = 8.03, *p* = .045. The condition × time × positive AO expectancies interaction effects at the six-month follow-up was statistically significant, *b* = 0.03, *p* = .009. When the condition × time interaction effects were examined among participants above and below the median of positive AO expectancies, the effects were not statistically significant, *d* = −0.29, *p* = .13, *d* = 0.15, *p* = .35. The supplementary file provides the results of the moderation analyses.

## 4 Discussion

This study investigated whether a web-based brief intervention could reduce problem drinking among Japanese adults and whether levels of AUD and AO expectancies would moderate the effects of the intervention. The intervention comprised an assessment of problem drinking, PNF, psychoeducation, and a short quiz. We implemented a two-armed group-parallel randomized controlled trial using two different crowdsourcing websites.

We rejected the primary null hypothesis as the intervention reduced the weekly drinking quantity at the two- and six-month follow-ups with small effect sizes: the between-group average reductions were 2.87 drinks and 1.94 drinks, respectively, when subtracted from the baseline and waitlist groups. This effect was weaker than those among Canadian adults with unhealthy alcohol use (Cunningham et al., 2009) but comparable to those among young Swiss adults with unhealthy alcohol use (Bertholet et al., 2015) and among university students with risky drinking in New Zealand (Kypri et al., 2014).

This study did not find the effect of the intervention on weekly abstinent days, largest drinking in one day, and alcohol-related consequences. The intervention effect may not be effective on weekly abstinent days, as Kypri et al. (2014) did not find intervention effects on drinking days among university students. The findings are also comparable to previous studies that did not find intervention effects on largest drinking quantity in one day and alcohol-related consequences (Bertholet et al., 2015, Cunningham, Godinho, & Kushnir, 2017). While these previous studies used different samples, findings suggest that an online brief intervention may have limited effects in reducing problematic drinking or its harmful effects in Japanese adults.

This study could not conclude that levels of AUD and AO expectancies were associated with the actual intervention effect on the measured outcomes. The intervention may have some effects across different levels of alcohol-related problems or beliefs, while more investigation is required to confirm this finding.

This study has several limitations. First, this study was underpowered due to high drop-out rate and inability to gather the appropriate number of participants determined by the initial power analysis. Additionally, a larger number of younger participants dropped out of this study. This demographic difference may have created a risk of bias in reported outcomes. Second, sampling may be an issue. As the recruitment took place in crowdsourcing websites, our sample may not be representative of the general Japanese population. While the crowdsourcing websites employed identification verification, which helped us ensure that one participant can provide only one response, fraudulent and duplicate responses by creating multiple accounts were technically possible. These are valid concerns when recruiting participants from crowdsourcing websites (Godinho, Cunningham, & Schell, 2020). Third, assigning a waitlist group may have created “readiness to change” expectancy and consequently inflated intervention effects (Cunningham, Kypri, & McCambridge, 2013). Fourth, as participants were unblinded at follow-ups, not providing monetary compensation at baseline and one-month follow-up may have been associated with dropout and lower treatment effect. However, it may have also helped respondents participate in subsequent follow-ups. Evidently, the treatment effect on weekly drinking quantity was only statistically non-significant at the one-month follow-up.

In conclusion, this study demonstrated the effects of a computerized intervention for reducing weekly drinking levels among Japanese who engage in problematic alcohol use. This finding is generally consistent with studies conducted among Western samples and suggests the generalizability and usefulness of computerized interventions among the Japanese population. The findings provide further support for computerized interventions as a viable option to help individuals with AUD reduce their drinking without face-to-face treatment.

## CRediT authorship contribution statement

**Toshitaka Hamamura:** Conceptualization, Investigation, Funding acquisition, Methodology, Data curation, Visualization, Writing – original draft. **Shinichiro Suganuma:** Investigation, Methodology. **Ayumi Takano:** Methodology. **Toshihiko Matsumoto:** Methodology. **Haruhiko Shimoyama:** Funding acquisition, Supervision, Investigation.

## Declaration of Competing Interest

The authors declare the following financial interests/personal relationships which may be considered as potential competing interests: This research was supported in part by grants from the Graduate Program for Social ICT Global Creative Leaders at the University of Tokyo. Toshitaka Hamamura reports personal fees from KDDI Research Inc. outside the submitted work.
