# Supplementary material for: The effectiveness of a web-based intervention for Japanese adults with problem drinking: An online randomized controlled trial
Source: Addict Behav Rep. 2021 Dec 14;15:100400. doi: 10.1016/j.abrep.2021.100400 (PMC8717418; doi:10.1016/j.abrep.2021.100400)
Supplement: Supplementary data 1 [file mmc1.docx]

# Supplementary file

## Table S1. Model comparison examining the three-way interaction effects

| Outcome and moderator | Model | *df* | χ^2^ | *p* | LL | AIC | BIC |
| --- | --- | --- | --- | --- | --- | --- | --- |
| Weekly drinking quantity |  |  |  |  |  |  |  |
| Baseline AUDIT | 1 | 24 |  |  | −1082.85 | 2213.70 | 2337.15 |
|  | 2 | 27 | 4.41 | .22 | −1080.65 | 2215.29 | 2354.17 |
| Positive AOE | 1 | 24 |  |  | −1169.71 | 2387.42 | 2511.07 |
|  | 2 | 27 | 1.19 | .76 | −1169.11 | 2392.23 | 2531.34 |
| Negative AOE | 1 | 24 |  |  | −1167.97 | 2383.94 | 2507.59 |
|  | 2 | 27 | 4.87 | .18 | −1165.53 | 2385.06 | 2524.17 |
| Weekly abstinent days |  |  |  |  |  |  |  |
| Baseline AUDIT | 1 | 24 |  |  | −1047.33 | 2142.66 | 2266.11 |
|  | 2 | 27 | 2.30 | .51 | −1046.18 | 2146.36 | 2285.24 |
| Positive AOE | 1 | 24 |  |  | −1087.03 | 2222.06 | 2345.71 |
|  | 2 | 27 | 2.56 | .47 | −1085.75 | 2225.50 | 2364.61 |
| Negative AOE | 1 | 24 |  |  | −1086.51 | 2221.03 | 2344.68 |
|  | 2 | 27 | 4.77 | .19 | −1084.13 | 2222.26 | 2361.38 |
| Largest drinking quantity in one day |  |  |  |  |  |  |  |
| Baseline AUDIT | 1 | 24 |  |  | −816.39 | 1680.79 | 1804.23 |
|  | 2 | 27 | 0.66 | .88 | −816.06 | 1686.12 | 1825.00 |
| Positive AOE | 1 | 24 |  |  | −838.84 | 1725.68 | 1849.34 |
|  | 2 | 27 | 3.37 | .34 | −837.16 | 1728.32 | 1867.43 |
| Negative AOE | 1 | 24 |  |  | −843.62 | 1735.24 | 1858.90 |
|  | 2 | 27 | 1.28 | .73 | −842.98 | 1739.96 | 1879.08 |
| Alcohol-related consequences |  |  |  |  |  |  |  |
| Baseline AUDIT | 1 | 24 |  |  | −962.52 | 1973.04 | 2096.49 |
|  | 2 | 27 | 6.06 | .11 | −959.49 | 1972.99 | 2111.87 |
| Positive AOE | 1 | 24 |  |  | −1037.92 | 2123.83 | 2247.49 |
|  | 2 | 27 | 8.03 | .045 | −1033.90 | 2121.80 | 2260.91 |
| Negative AOE | 1 | 24 |  |  | −1015.44 | 2078.88 | 2202.53 |
|  | 2 | 27 | 0.56 | .91 | −1015.16 | 2084.32 | 2223.43 |

*Note.* Model 1 includes the main effects and two-way interaction effects of condition, time and the moderator. Model 2 includes the condition × time × moderator interaction effect in addition to the effects in Model 1. AIC = Akaike information criterion. AOE = Alcohol outcome expectancies. AUDIT = Alcohol use disorder identification test. BIC = Bayesian information criterion. LL = Log likelihood.

## Table S2. Interaction effects on alcohol-related consequences at the six-month follow-up examining the moderation effects

| Fixed effects | *b* | *SE* | *df* | *t* | *p* | *d* | 95% CI |
| --- | --- | --- | --- | --- | --- | --- | --- |
| Condition × Time × Positive AOE | 0.03 | 0.01 | 719 | 2.63 | .009 | -- | -- |
| Among high positive AOE |  |  |  |  |  |  |  |
| Condition × Time | −0.17 | 0.11 | 340 | −1.54 | .13 | −0.29 | −0.65, 0.08 |
| Among low positive AOE |  |  |  |  |  |  |  |
| Condition × Time | 0.10 | 0.11 | 379 | 0.93 | .35 | 0.15 | −0.17, 0.47 |

*Note.* AOE = Alcohol outcome expectancies. CI = Confidence interval. SE = Standard error.


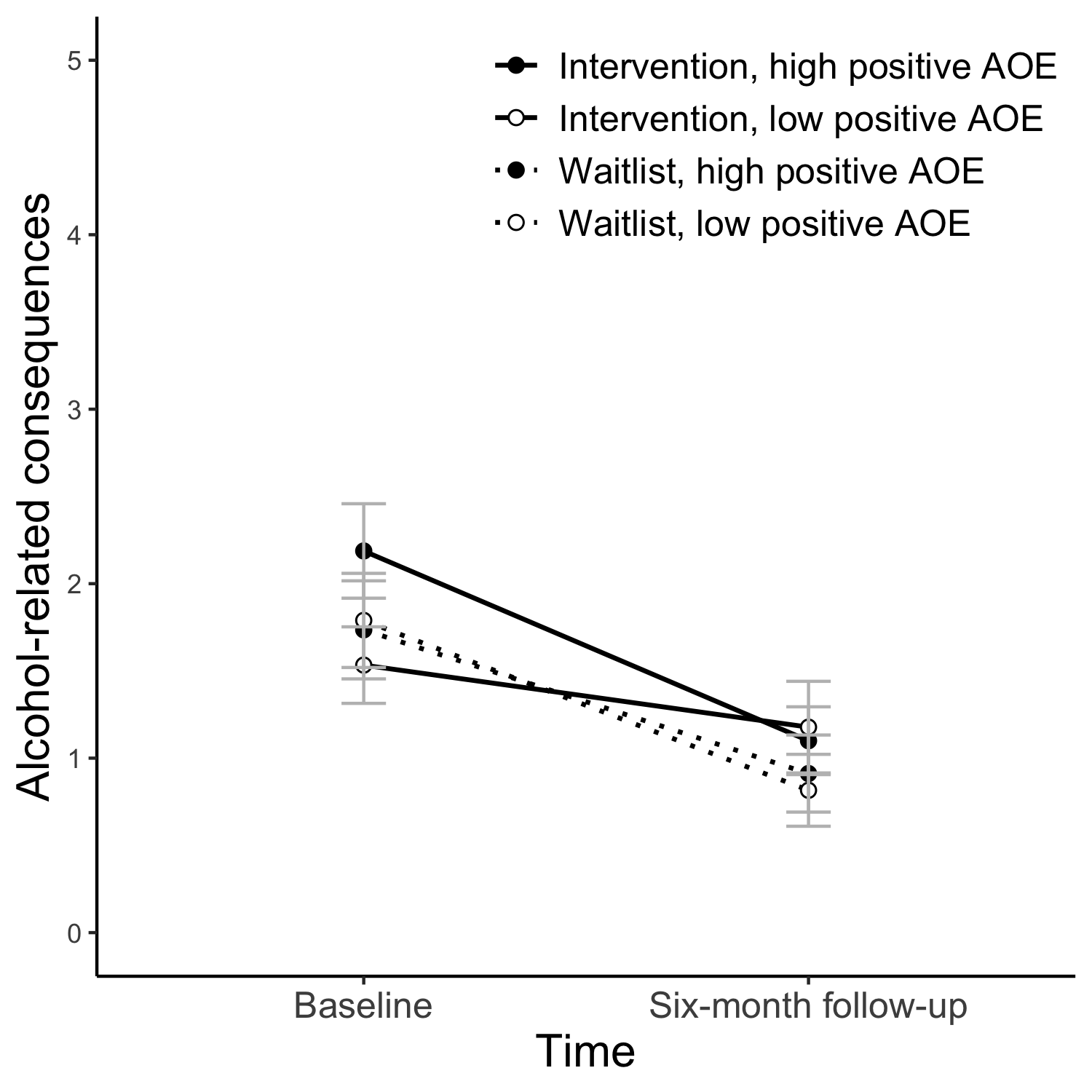


## Figure S1. Simple slope analysis of participants’ alcohol-related consequences by positive alcohol outcome (AO) expectancies at the baseline and six-month follow-up. Dots and error bars represent the mean and standard error of the alcohol-related consequences, respectively.
