# Supplementary material for: The effectiveness of a web-based intervention for Japanese adults with problem drinking: An online randomized controlled trial
Source: Addict Behav Rep. 2021 Dec 14;15:100400. doi: 10.1016/j.abrep.2021.100400 (PMC8717418; doi:10.1016/j.abrep.2021.100400)
Supplement: Supplementary data 2 [file mmc2.zip › analysis_output.html]

output


# output

#### Toshitaka Hamamura

#### 12/19/2021

```
knitr::opts_chunk$set(echo = TRUE)
```

# 1 Cleaning the data

```
#load('RData/cleaning_ppa.RData')
main <- read.csv("data/20180814_raw_main.csv")
# name variables ----
main$condition <- factor(main$condition,
                      levels = c(1,2),
                      labels = c("Intervention","Waitlist"))
main$time <- factor(main$time,
                 levels = c(1,2,3,4),
                 labels =  c("baseline","one_month","two_months","six_months"))
main$sex <- factor(main$sex,
                levels = c(1,2),
                labels = c("men","women"))
# modify ----
main$ddq_mon[which(main$ddq_mon=='30+')] <- '30'
main$ddq_tue[which(main$ddq_tue=='30+')] <- '30'
main$ddq_wed[which(main$ddq_wed=='30+')] <- '30'
main$ddq_thu[which(main$ddq_thu=='30+')] <- '30'
main$ddq_fri[which(main$ddq_fri=='30+')] <- '30'
main$ddq_sat[which(main$ddq_sat=='30+')] <- '30'
main$ddq_sun[which(main$ddq_sun=='30+')] <- '30'
main$most_drink[which(main$most_drink=='30+')] <- '30'
# add_variables ---- 
main$ddf_mon <- ifelse(main$ddq_mon>=1,main$ddf_mon <- 1,main$ddf_mon <- 0)
main$ddf_tue <- ifelse(main$ddq_tue>=1,main$ddf_tue <- 1,main$ddf_tue <- 0)
main$ddf_wed <- ifelse(main$ddq_wed>=1,main$ddf_wed <- 1,main$ddf_wed <- 0)
main$ddf_thu <- ifelse(main$ddq_thu>=1,main$ddf_thu <- 1,main$ddf_thu <- 0)
main$ddf_fri <- ifelse(main$ddq_fri>=1,main$ddf_fri <- 1,main$ddf_fri <- 0)
main$ddf_sat <- ifelse(main$ddq_sat>=1,main$ddf_sat <- 1,main$ddf_sat <- 0)
main$ddf_sun <- ifelse(main$ddq_sun>=1,main$ddf_sun <- 1,main$ddf_sun <- 0)
# make_numeric ----
num_col <- c("id", "age", "height", "weight",
             "ae1", "ae2", "ae3", "ae4", "ae5", "ae6", "ae7", "ae8", "ae9", "ae10",
             "ae11", "ae12", "ae13", "ae14", "ae15", "ae16", "ae17", "ae18", "ae19", "ae20", 
             "audit1", "audit2", "audit3", "audit4", "audit5", "audit6", "audit7", "audit8", "audit9", "audit10",
             "most_drink", "ddq_mon", "ddq_tue", "ddq_wed", "ddq_thu", "ddq_fri", "ddq_sat", "ddq_sun",
             "consq_rltnshp", "consq_health", "consq_satisf", "consq_famly", "consq_work", "consq_finance")
main[num_col] <- sapply(main[num_col], as.numeric)
# convert audit3 to the standard item response ----
main$audit3[main$audit3==3] <- 2
main$audit3[main$audit3==4 | main$audit3==5] <- 3
main$audit3[main$audit3==6] <- 4
# sum_variables ----
main$ae_positive <- rowSums(main[c("ae1", "ae3", "ae5", "ae8", "ae10", "ae11", "ae12", "ae15", "ae17", "ae19")])
main$ae_negative <- rowSums(main[c("ae2", "ae4", "ae6", "ae7", "ae9", "ae13", "ae14", "ae16", "ae18", "ae20")])
main$ddq <- main$ddq_mon + main$ddq_tue + main$ddq_wed + main$ddq_thu + main$ddq_fri + main$ddq_sat + main$ddq_sun
main$ddf <- main$ddf_mon + main$ddf_tue + main$ddf_wed + main$ddf_thu + main$ddf_fri + main$ddf_sat + main$ddf_sun
main$audit <- rowSums(main[c("audit1","audit2","audit3","audit4","audit5","audit6","audit7","audit8","audit9","audit10")])#audit score
main$consq <- main$consq_rltnshp + main$consq_health + main$consq_satisf + main$consq_famly + main$consq_work + main$consq_finance
# delete_rows ----
df <- main # 651 participants
df <- df[-which(df$valid_user==9),] #drop out before allocation: 1476 -> 1439 (37 responses)
df <- df[!df$id %in% c(1:24,121:126),]# invalid entries: test responses: 1439 -> 1397 (42 responses)
df <- df[-which(df$valid_response==9 & df$id %in% c(160, 207, 334, 554, 560, 605)),] #invalid  entries: other (1397 -> 1388 = 9 responses, 591 participants left) 
id_audit <- df$id[which(df$audit<8 & df$time=="baseline")] # audit < 8 
df <- df[-which(df$id %in% id_audit),] # audit < 8, 1388 -> 1278 (110 responses, 45 participants )
# add_NA_in_dataframe ----
df <- df[order(df$time),]
df <- df[order(df$id),]
d <- df # for scale reliability
w <- reshape(df[, c("ae_negative", "ae_positive", "age", "audit", "condition", 
                    "consq", "ddf", "ddq", "id", "most_drink", "sex", "time")],
             v.names = c("audit", "consq","ddf", "ddq", "most_drink"),
             timevar = "time",
             idvar = "id",
             direction = "wide")
w$audit_baseline <- w$audit.baseline
w <- subset(w, select = c(id, condition, age, sex, ae_positive, ae_negative, audit_baseline, audit.baseline, audit.one_month, audit.two_months, audit.six_months, ddq.baseline, ddq.one_month, ddq.two_months, ddq.six_months, ddf.baseline, ddf.one_month, ddf.two_months, ddf.six_months, most_drink.baseline, most_drink.one_month, most_drink.two_months, most_drink.six_months, consq.baseline, consq.one_month, consq.two_months, consq.six_months))
# long_format ----
df <- reshape(w[, c("ae_negative", "ae_positive", "age", "audit_baseline", "condition", "id", "sex", 
                    "audit.baseline", "consq.baseline", "ddf.baseline", "ddq.baseline", "most_drink.baseline",
                    "audit.one_month", "consq.one_month", "ddf.one_month", "ddq.one_month", "most_drink.one_month",
                    "audit.two_months", "consq.two_months", "ddf.two_months", "ddq.two_months", "most_drink.two_months",
                    "audit.six_months", "consq.six_months", "ddf.six_months", "ddq.six_months", "most_drink.six_months")],
              varying = c("audit.baseline", "consq.baseline", "ddf.baseline", "ddq.baseline", "most_drink.baseline",
                          "audit.one_month", "consq.one_month", "ddf.one_month", "ddq.one_month", "most_drink.one_month",
                          "audit.two_months", "consq.two_months", "ddf.two_months", "ddq.two_months", "most_drink.two_months",
                          "audit.six_months", "consq.six_months", "ddf.six_months", "ddq.six_months", "most_drink.six_months"),
              v.names = c("audit", "consq", "ddf", "ddq", "most_drink"),
              timevar = "time",
              idvar = "id",
              direction = "long")
df$time <- factor(df$time,
                  levels = c(1,2,3,4),
                  labels =  c("baseline","one_month","two_months","six_months"))
df <- subset(df, select = c(id, condition, time, age, sex, ae_positive, ae_negative, audit, audit_baseline, ddq, ddf, most_drink, consq))
# Weekly abstinent days ----

d$dfd <- 7-d$ddf
# factor ----
w$sex <- as.factor(w$sex)
df$ddq_log <- log(df$ddq+1)
df$dfd <- 7-df$ddf
df$dfd_log <- log(df$dfd+1)
df$most_drink_log <- log(df$most_drink+1)
df$consq_log <- log(df$consq+1)
# median_split ----
df_high_audit <- df[which(df$audit_baseline<median(df$audit_baseline, na.rm = TRUE)),]
df_low_audit <- df[which(df$audit_baseline>=median(df$audit_baseline, na.rm = TRUE)),]
df_high_paoe <- df[which(df$ae_positive<median(df$ae_positive)),]
df_low_paoe <- df[which(df$ae_positive>=median(df$ae_positive)),]
df_high_naoe <- df[which(df$ae_negative<median(df$ae_negative)),]
df_low_naoe <- df[which(df$ae_negative>=median(df$ae_negative)),]
```

# 2 Power analysis

```
library(pwr)
pwr.anova.test(k = 2, n = , f = 0.1, sig.level = 0.05, power = 0.80)
```

```
## 
##      Balanced one-way analysis of variance power calculation 
## 
##               k = 2
##               n = 393.4057
##               f = 0.1
##       sig.level = 0.05
##           power = 0.8
## 
## NOTE: n is number in each group
```

# 3 Attrition

```
library(dplyr)
library(janitor)
knitr::kable(length(d$id[which(d$time=="baseline")]), caption = "Number of participants at baseline (5 more participants enrolled but missing)") # delete
```

Number of participants at baseline (5 more participants enrolled but missing)

| x |
| --- |
| 541 |

```
knitr::kable(length(w$id)/1250*100, caption = "Includesion rate")
```

Includesion rate

| x |
| --- |
| 43.68 |

```
knitr::kable(w %>% tabyl(sex), digits = 4)
```

| sex | n | percent |
| --- | --- | --- |
| men | 310 | 0.5678 |
| women | 236 | 0.4322 |

```
# attrition ----
knitr::kable(table(d$time), digits = 4, caption = "Responses at each time point")
```

Responses at each time point

| Var1 | Freq |
| --- | --- |
| baseline | 541 |
| one\_month | 289 |
| two\_months | 270 |
| six\_months | 178 |

```
knitr::kable(table(d$time)/table(df$time), digits = 4, caption = "Response rates at each time point")
```

Response rates at each time point

| Var1 | Freq |
| --- | --- |
| baseline | 0.9908 |
| one\_month | 0.5293 |
| two\_months | 0.4945 |
| six\_months | 0.3260 |

```
knitr::kable(table(d$condition, d$time), digits = 4, caption = "Responses in each condition and time. (5 responses are missing at the baseline.)")
```

Responses in each condition and time. (5 responses are missing at the baseline.)

|  | baseline | one\_month | two\_months | six\_months |
| --- | --- | --- | --- | --- |
| Intervention | 264 | 150 | 142 | 98 |
| Waitlist | 277 | 139 | 128 | 80 |

```
knitr::kable(table(d$condition, d$time)/table(df$condition, df$time), digits = 4, caption = "Response rates in each condition and time.")
```

Response rates in each condition and time.

|  | baseline | one\_month | two\_months | six\_months |
| --- | --- | --- | --- | --- |
| Intervention | 0.9888 | 0.5618 | 0.5318 | 0.3670 |
| Waitlist | 0.9928 | 0.4982 | 0.4588 | 0.2867 |

```
knitr::kable(table(df$condition, df$time) - table(d$condition, d$time), caption = "Figure 1: Lost to follow-up") # 5 responses are missing at the baseline
```

Figure 1: Lost to follow-up

|  | baseline | one\_month | two\_months | six\_months |
| --- | --- | --- | --- | --- |
| Intervention | 3 | 117 | 125 | 169 |
| Waitlist | 2 | 140 | 151 | 199 |

```
knitr::kable(1- table(d$condition, d$time)/table(df$condition, df$time), digits = 4, caption = "Figure 1: Lost to follow-up (%)")
```

Figure 1: Lost to follow-up (%)

|  | baseline | one\_month | two\_months | six\_months |
| --- | --- | --- | --- | --- |
| Intervention | 0.0112 | 0.4382 | 0.4682 | 0.6330 |
| Waitlist | 0.0072 | 0.5018 | 0.5412 | 0.7133 |

```
# demographc_differencs ----
w$missing <- 0
w$missing[which(is.na(w$ddq.one_month))] <- 1
w$missing[which(is.na(w$ddq.two_month))] <- 1
w$missing[which(is.na(w$ddq.six_months))] <- 1
fit_miss <- manova(cbind(w$sex, w$age) ~ w$missing)
summary(fit_miss, test = "Wilks")
```

```
##            Df   Wilks approx F num Df den Df    Pr(>F)    
## w$missing   1 0.96937   8.5783      2    543 0.0002148 ***
## Residuals 544                                             
## ---
## Signif. codes:  0 '***' 0.001 '**' 0.01 '*' 0.05 '.' 0.1 ' ' 1
```

```
summary.aov(fit_miss)
```

```
##  Response 1 :
##              Df  Sum Sq Mean Sq F value Pr(>F)
## w$missing     1   0.132 0.13164   0.535 0.4648
## Residuals   544 133.861 0.24607               
## 
##  Response 2 :
##              Df Sum Sq Mean Sq F value    Pr(>F)    
## w$missing     1   1816 1815.97  16.945 4.445e-05 ***
## Residuals   544  58299  107.17                      
## ---
## Signif. codes:  0 '***' 0.001 '**' 0.01 '*' 0.05 '.' 0.1 ' ' 1
```

```
## eta_squared_sex ----
0.132/(0.132+133.861)
```

```
## [1] 0.0009851261
```

```
## eta_squared_age ----
1816/(1816+58299)
```

```
## [1] 0.03020877
```

# 4 Descriptive statistics

```
library(dplyr)
library(tidyr)
id <- d %>% 
  mutate(time = factor(time, labels = c("Baseline","One-month follow-up","Two-month follow-up","Six-month follow-up")))
id_outcome <- id %>% 
  mutate(condition = factor(condition, labels = c("Intervention", "Waitlist")))
# audit_baseline ----
id_audit <- id_outcome %>%
  group_by(condition, time) %>%
  summarise(n = n(),
            Mean = mean(audit),
            SD = sd(audit),
            Median = median(audit)
            )
knitr::kable(id_audit, digits = 2, caption = "AUDIT")
```

AUDIT

| condition | time | n | Mean | SD | Median |
| --- | --- | --- | --- | --- | --- |
| Intervention | Baseline | 264 | 13.89 | 5.22 | 12.0 |
| Intervention | One-month follow-up | 150 | 11.37 | 5.45 | 10.0 |
| Intervention | Two-month follow-up | 142 | 9.77 | 5.78 | 9.0 |
| Intervention | Six-month follow-up | 98 | 8.69 | 5.74 | 7.5 |
| Waitlist | Baseline | 277 | 14.14 | 5.35 | 13.0 |
| Waitlist | One-month follow-up | 139 | 11.63 | 5.19 | 11.0 |
| Waitlist | Two-month follow-up | 128 | 10.04 | 5.74 | 9.0 |
| Waitlist | Six-month follow-up | 80 | 8.51 | 5.42 | 8.0 |

```
# ae_positive ----
id_ae_positive <- id_outcome %>%
  group_by(condition) %>%
  summarise(n = n(),
            Mean = mean(ae_positive),
            SD = sd(ae_positive),
            Median = median(ae_positive)
            )
knitr::kable(id_ae_positive, digits = 2, caption = "Positive AOE")
```

Positive AOE

| condition | n | Mean | SD | Median |
| --- | --- | --- | --- | --- |
| Intervention | 654 | 42.18 | 7.14 | 43 |
| Waitlist | 624 | 41.47 | 7.49 | 42 |

```
# ae_negative ----
id_ae_negative <- id_outcome %>%
  group_by(condition) %>%
  summarise(n = n(),
            Mean = mean(ae_negative),
            SD = sd(ae_negative),
            Median = median(ae_negative)
            )
knitr::kable(id_ae_negative, digits = 2, caption = "Negative AOE")
```

Negative AOE

| condition | n | Mean | SD | Median |
| --- | --- | --- | --- | --- |
| Intervention | 654 | 23.29 | 7.08 | 23 |
| Waitlist | 624 | 24.04 | 6.70 | 23 |

```
# drinking_quantity ----
id_ddq <- id_outcome %>%
  group_by(condition, time) %>%
  summarise(n = n(),
            Mean = mean(ddq),
            SD = sd(ddq),
            Median = median(ddq)
            )
knitr::kable(id_ddq, digits = 2, caption = "Weekly drinking quantity")
```

Weekly drinking quantity

| condition | time | n | Mean | SD | Median |
| --- | --- | --- | --- | --- | --- |
| Intervention | Baseline | 264 | 21.06 | 16.14 | 16.5 |
| Intervention | One-month follow-up | 150 | 14.51 | 10.64 | 13.0 |
| Intervention | Two-month follow-up | 142 | 14.77 | 12.54 | 12.0 |
| Intervention | Six-month follow-up | 98 | 13.65 | 12.57 | 11.0 |
| Waitlist | Baseline | 277 | 20.78 | 15.26 | 17.0 |
| Waitlist | One-month follow-up | 139 | 16.37 | 12.54 | 14.0 |
| Waitlist | Two-month follow-up | 128 | 17.37 | 14.95 | 13.0 |
| Waitlist | Six-month follow-up | 80 | 15.70 | 14.19 | 10.0 |

```
# Weekly abstinent days ----
id_dfd <- id_outcome %>%
  group_by(condition, time) %>%
  summarise(n = n(),
            Mean = mean(dfd),
            SD = sd(dfd),
            Median = median(dfd)
            )
knitr::kable(id_dfd, digits = 2, caption = "Weekly abstinent days")
```

Weekly abstinent days

| condition | time | n | Mean | SD | Median |
| --- | --- | --- | --- | --- | --- |
| Intervention | Baseline | 264 | 1.55 | 1.89 | 0 |
| Intervention | One-month follow-up | 150 | 2.21 | 2.17 | 2 |
| Intervention | Two-month follow-up | 142 | 2.46 | 2.30 | 2 |
| Intervention | Six-month follow-up | 98 | 2.50 | 2.38 | 2 |
| Waitlist | Baseline | 277 | 1.50 | 1.81 | 1 |
| Waitlist | One-month follow-up | 139 | 1.97 | 2.11 | 1 |
| Waitlist | Two-month follow-up | 128 | 2.19 | 2.26 | 2 |
| Waitlist | Six-month follow-up | 80 | 1.96 | 2.21 | 1 |

```
# most_drink ----
id_most_drink <- id_outcome %>%
  group_by(condition, time) %>%
  summarise(n = n(),
            Mean = mean(most_drink),
            SD = sd(most_drink),
            Median = median(most_drink)
            )
knitr::kable(id_most_drink, digits = 2, caption = "Largest drinking quantity in one day")
```

Largest drinking quantity in one day

| condition | time | n | Mean | SD | Median |
| --- | --- | --- | --- | --- | --- |
| Intervention | Baseline | 264 | 9.77 | 5.63 | 8.0 |
| Intervention | One-month follow-up | 150 | 6.48 | 4.02 | 5.5 |
| Intervention | Two-month follow-up | 142 | 5.90 | 3.79 | 5.0 |
| Intervention | Six-month follow-up | 98 | 5.41 | 4.47 | 4.0 |
| Waitlist | Baseline | 277 | 10.34 | 5.73 | 10.0 |
| Waitlist | One-month follow-up | 139 | 6.78 | 3.79 | 6.0 |
| Waitlist | Two-month follow-up | 128 | 6.53 | 3.88 | 6.0 |
| Waitlist | Six-month follow-up | 80 | 6.46 | 5.42 | 5.5 |

```
# consq ----
id_consq <- id_outcome %>%
  group_by(condition, time) %>%
  summarise(n = n(),
            Mean = mean(consq),
            SD = sd(consq),
            Median = median(consq)
            )
knitr::kable(id_consq, digits = 2, caption = "Alcohol-related consequences")
```

Alcohol-related consequences

| condition | time | n | Mean | SD | Median |
| --- | --- | --- | --- | --- | --- |
| Intervention | Baseline | 264 | 2.02 | 1.76 | 2 |
| Intervention | One-month follow-up | 150 | 1.09 | 1.45 | 1 |
| Intervention | Two-month follow-up | 142 | 1.06 | 1.59 | 0 |
| Intervention | Six-month follow-up | 98 | 1.10 | 1.55 | 0 |
| Waitlist | Baseline | 277 | 1.88 | 1.71 | 1 |
| Waitlist | One-month follow-up | 139 | 0.93 | 1.31 | 0 |
| Waitlist | Two-month follow-up | 128 | 0.88 | 1.31 | 0 |
| Waitlist | Six-month follow-up | 80 | 0.90 | 1.37 | 0 |

# 5 Visualization

```
library(dplyr)
library(tidyr)
library(ggplot2)
```

```
## Warning: package 'ggplot2' was built under R version 4.1.1
```

```
# set_up ----
id <- d %>% 
  mutate(time = factor(time, labels = c("1_baseline","2_one_month","3_two_months","4_six_months")))
id_outcome <- id %>% 
  mutate(condition = factor(condition, labels = c("Intervention", "Waitlist")))
id_ddq <- id_outcome %>%
  group_by(condition, time) %>%
  summarise(n = n(),
            mean = mean(ddq),
            sd = sd(ddq),
            se = sd/sqrt(n)
            )
```

```
## `summarise()` has grouped output by 'condition'. You can override using the `.groups` argument.
```

```
# box_plot ----
ggplot(df, aes(x = time, y = ddq, fill = condition)) + 
  theme_classic() + 
  geom_boxplot() + 
  labs(x = "Time", y = "Weekly drinking quantity") + 
  scale_x_discrete(labels = c('Baseline','One Month','Two Months', 'Six Months')) + 
  scale_y_continuous(minor_breaks = seq(0, 108, 10), breaks = seq(0, 108, 10)) + 
  scale_fill_manual(values = c("white", "darkgray")) + 
  theme(
      legend.text = element_text(size = 12),
    axis.title.x = element_text(size = 15),
    axis.text.x = element_text(size = 12),
    axis.title = element_text(size = 15),
    legend.title = element_blank(),
    legend.justification=c(1.9,-0.1),
    legend.position=c(1.2,0.8)
    ) + 
  expand_limits(y=108:0)
```

```
## Warning: Removed 907 rows containing non-finite values (stat_boxplot).
```

# 6 Scale reliability

```
##           audit_baseline
## alpha          0.6983131
## alpha.ord      0.7415919
## omega          0.7045531
## omega2         0.7045531
## omega3         0.7239307
## avevar         0.2791140
```

```
##        aoe_positive
## alpha     0.8552855
## omega     0.8477961
## omega2    0.8477961
## omega3    0.8171034
## avevar    0.3628748
```

```
##        aoe_negative
## alpha     0.7979495
## omega     0.7675318
## omega2    0.7675318
## omega3    0.6654055
## avevar    0.2849397
```

```
##        drinking_quantity_baseline
## alpha                   0.8907048
## omega                   0.8870409
## omega2                  0.8870409
## omega3                  0.8739409
## avevar                  0.5295025
```

```
##        drinking_quantity_one_month
## alpha                    0.8900343
## omega                    0.8867680
## omega2                   0.8867680
## omega3                   0.8731516
## avevar                   0.5297891
```

```
##        drinking_quantity_two_month
## alpha                    0.8959391
## omega                    0.8932858
## omega2                   0.8932858
## omega3                   0.8803149
## avevar                   0.5468063
```

```
##        drinking_quantity_six_month
## alpha                    0.9298248
## omega                    0.9285395
## omega2                   0.9285395
## omega3                   0.9210911
## avevar                   0.6508143
```

```
##           abstinent_days_baseline
## alpha                   0.7775756
## alpha.ord               0.8835839
## omega                   0.8105594
## omega2                  0.8105594
## omega3                  0.8148614
## avevar                  0.5575401
```

```
##           abstinent_days_one_month
## alpha                    0.8134121
## alpha.ord                0.9067775
## omega                    0.8408325
## omega2                   0.8408325
## omega3                   0.8472743
## avevar                   0.6129801
```

```
##           abstinent_days_two_month
## alpha                    0.8376922
## alpha.ord                0.9264231
## omega                    0.8590756
## omega2                   0.8590756
## omega3                   0.8673391
## avevar                   0.6678272
```

```
##           abstinent_days_six_month
## alpha                    0.8509635
## alpha.ord                0.9357877
## omega                    0.8741308
## omega2                   0.8741308
## omega3                   0.8862196
## avevar                   0.7036487
```

```
##           consq_baseline
## alpha          0.7018299
## alpha.ord      0.8403322
## omega          0.7109852
## omega2         0.7109852
## omega3         0.7141024
## avevar         0.4854828
```

```
##           consq_one_month
## alpha           0.6979790
## alpha.ord       0.8746662
## omega           0.7158631
## omega2          0.7158631
## omega3          0.7212411
## avevar          0.5560503
```

```
##           consq_two_month
## alpha           0.7538457
## alpha.ord       0.9065471
## omega           0.7644986
## omega2          0.7644986
## omega3          0.7667515
## avevar          0.6339618
```

```
##           consq_six_month
## alpha           0.7486393
## alpha.ord       0.9002415
## omega           0.7671474
## omega2          0.7671474
## omega3          0.7786672
## avevar          0.6347413
```

# 7 Intervention effects

```
##                                  p-value cohen_d  LCL_d
## (Intercept)                        0.000   4.241  4.121
## conditionWaitlist                  0.980  -0.002 -0.171
## timeone_month                      0.000  -0.520 -0.651
## timetwo_months                     0.000  -0.688 -0.852
## timesix_months                     0.000  -0.919 -1.115
## conditionWaitlist:timeone_month    0.202   0.124 -0.066
## conditionWaitlist:timetwo_months   0.025   0.275  0.035
## conditionWaitlist:timesix_months   0.022   0.338  0.049
```

Weekly drinking quantity

|  | Value | Std.Error | DF | t-value | p-value | cohen\_d | LCL\_d | UCL\_d |
| --- | --- | --- | --- | --- | --- | --- | --- | --- |
| (Intercept) | 2.866 | 0.042 | 725 | 68.978 | 0.000 | 4.241 | 4.121 | 4.362 |
| conditionWaitlist | -0.001 | 0.058 | 544 | -0.025 | 0.980 | -0.002 | -0.171 | 0.166 |
| timeone\_month | -0.351 | 0.045 | 725 | -7.761 | 0.000 | -0.520 | -0.651 | -0.389 |
| timetwo\_months | -0.465 | 0.057 | 725 | -8.190 | 0.000 | -0.688 | -0.852 | -0.523 |
| timesix\_months | -0.621 | 0.067 | 725 | -9.237 | 0.000 | -0.919 | -1.115 | -0.724 |
| conditionWaitlist:timeone\_month | 0.084 | 0.066 | 725 | 1.276 | 0.202 | 0.124 | -0.066 | 0.314 |
| conditionWaitlist:timetwo\_months | 0.186 | 0.083 | 725 | 2.247 | 0.025 | 0.275 | 0.035 | 0.514 |
| conditionWaitlist:timesix\_months | 0.229 | 0.100 | 725 | 2.289 | 0.022 | 0.338 | 0.049 | 0.628 |

```
##                                  p-value cohen_d  LCL_d
## (Intercept)                        0.000   0.938  0.818
## conditionWaitlist                  0.808  -0.021 -0.189
## timeone_month                      0.000   0.284  0.173
## timetwo_months                     0.000   0.428  0.299
## timesix_months                     0.000   0.469  0.300
## conditionWaitlist:timeone_month    0.582  -0.045 -0.206
## conditionWaitlist:timetwo_months   0.137  -0.143 -0.332
## conditionWaitlist:timesix_months   0.089  -0.217 -0.468
```

Weekly drinking frequency


|  | Value | Std.Error | DF | t-value | p-value | cohen\_d | LCL\_d | UCL\_d |
| --- | --- | --- | --- | --- | --- | --- | --- | --- |
| (Intercept) | 0.672 | 0.044 | 725 | 15.261 | 0.000 | 0.938 | 0.818 | 1.059 |
| conditionWaitlist | -0.015 | 0.062 | 544 | -0.244 | 0.808 | -0.021 | -0.189 | 0.148 |
| timeone\_month | 0.204 | 0.041 | 725 | 5.005 | 0.000 | 0.284 | 0.173 | 0.396 |
| timetwo\_months | 0.307 | 0.047 | 725 | 6.469 | 0.000 | 0.428 | 0.299 | 0.558 |
| timesix\_months | 0.336 | 0.062 | 725 | 5.445 | 0.000 | 0.469 | 0.300 | 0.638 |
| conditionWaitlist:timeone\_month | -0.032 | 0.059 | 725 | -0.551 | 0.582 | -0.045 | -0.206 | 0.116 |
| conditionWaitlist:timetwo\_months | -0.103 | 0.069 | 725 | -1.489 | 0.137 | -0.143 | -0.332 | 0.045 |
| conditionWaitlist:timesix\_months | -0.156 | 0.091 | 725 | -1.703 | 0.089 | -0.217 | -0.468 | 0.033 |

```
##                                  p-value cohen_d  LCL_d
## (Intercept)                        0.000   4.723  4.603
## conditionWaitlist                  0.216   0.107 -0.062
## timeone_month                      0.000  -0.782 -0.955
## timetwo_months                     0.000  -1.003 -1.183
## timesix_months                     0.000  -1.244 -1.477
## conditionWaitlist:timeone_month    0.902   0.016 -0.233
## conditionWaitlist:timetwo_months   0.359   0.122 -0.139
## conditionWaitlist:timesix_months   0.097   0.293 -0.052
```

Largested drinking quantity in one day

|  | Value | Std.Error | DF | t-value | p-value | cohen\_d | LCL\_d | UCL\_d |
| --- | --- | --- | --- | --- | --- | --- | --- | --- |
| (Intercept) | 2.260 | 0.029 | 725 | 76.809 | 0.000 | 4.723 | 4.603 | 4.844 |
| conditionWaitlist | 0.051 | 0.041 | 544 | 1.240 | 0.216 | 0.107 | -0.062 | 0.275 |
| timeone\_month | -0.374 | 0.042 | 725 | -8.876 | 0.000 | -0.782 | -0.955 | -0.609 |
| timetwo\_months | -0.480 | 0.044 | 725 | -10.906 | 0.000 | -1.003 | -1.183 | -0.822 |
| timesix\_months | -0.595 | 0.057 | 725 | -10.460 | 0.000 | -1.244 | -1.477 | -1.011 |
| conditionWaitlist:timeone\_month | 0.007 | 0.061 | 725 | 0.123 | 0.902 | 0.016 | -0.233 | 0.264 |
| conditionWaitlist:timetwo\_months | 0.059 | 0.064 | 725 | 0.918 | 0.359 | 0.122 | -0.139 | 0.384 |
| conditionWaitlist:timesix\_months | 0.140 | 0.084 | 725 | 1.663 | 0.097 | 0.293 | -0.052 | 0.639 |

```
##                                  p-value cohen_d  LCL_d
## (Intercept)                        0.000   1.470  1.349
## conditionWaitlist                  0.349  -0.081 -0.249
## timeone_month                      0.000  -0.545 -0.694
## timetwo_months                     0.000  -0.637 -0.796
## timesix_months                     0.000  -0.587 -0.754
## conditionWaitlist:timeone_month    0.541  -0.067 -0.280
## conditionWaitlist:timetwo_months   0.968  -0.005 -0.234
## conditionWaitlist:timesix_months   0.760  -0.038 -0.285
```

Alcohol-related consequences


|  | Value | Std.Error | DF | t-value | p-value | cohen\_d | LCL\_d | UCL\_d |
| --- | --- | --- | --- | --- | --- | --- | --- | --- |
| (Intercept) | 0.921 | 0.039 | 725 | 23.898 | 0.000 | 1.470 | 1.349 | 1.590 |
| conditionWaitlist | -0.050 | 0.054 | 544 | -0.937 | 0.349 | -0.081 | -0.249 | 0.088 |
| timeone\_month | -0.341 | 0.048 | 725 | -7.169 | 0.000 | -0.545 | -0.694 | -0.396 |
| timetwo\_months | -0.399 | 0.051 | 725 | -7.845 | 0.000 | -0.637 | -0.796 | -0.478 |
| timesix\_months | -0.368 | 0.053 | 725 | -6.895 | 0.000 | -0.587 | -0.754 | -0.420 |
| conditionWaitlist:timeone\_month | -0.042 | 0.068 | 725 | -0.611 | 0.541 | -0.067 | -0.280 | 0.147 |
| conditionWaitlist:timetwo\_months | -0.003 | 0.073 | 725 | -0.040 | 0.968 | -0.005 | -0.234 | 0.225 |
| conditionWaitlist:timesix\_months | -0.024 | 0.079 | 725 | -0.305 | 0.760 | -0.038 | -0.285 | 0.208 |

# 8 Moderation analysis

Moderation of AUDIT on weekly drinking quantity

|  | Model | df | AIC | BIC | logLik | Test | L.Ratio | p-value |
| --- | --- | --- | --- | --- | --- | --- | --- | --- |
| fit\_ddq\_log\_audit3 | 1 | 24 | 2213.703 | 2337.150 | -1082.852 |  | NA | NA |
| fit\_ddq\_log\_audit4 | 2 | 27 | 2215.294 | 2354.172 | -1080.647 | 1 vs 2 | 4.409 | 0.221 |

Moderation of positive AOE on weekly drinking quantity

|  | Model | df | AIC | BIC | logLik | Test | L.Ratio | p-value |
| --- | --- | --- | --- | --- | --- | --- | --- | --- |
| fit\_ddq\_log\_ae\_positive3 | 1 | 24 | 2387.416 | 2511.070 | -1169.708 |  | NA | NA |
| fit\_ddq\_log\_ae\_positive4 | 2 | 27 | 2392.228 | 2531.339 | -1169.114 | 1 vs 2 | 1.188 | 0.756 |

Moderation of negative AOE on weekly drinking quantity

|  | Model | df | AIC | BIC | logLik | Test | L.Ratio | p-value |
| --- | --- | --- | --- | --- | --- | --- | --- | --- |
| fit\_ddq\_log\_ae\_negative3 | 1 | 24 | 2383.936 | 2507.590 | -1167.968 |  | NA | NA |
| fit\_ddq\_log\_ae\_negative4 | 2 | 27 | 2385.062 | 2524.173 | -1165.531 | 1 vs 2 | 4.874 | 0.181 |

Moderation of AUDIT on weekly drinking frequency

|  | Model | df | AIC | BIC | logLik | Test | L.Ratio | p-value |
| --- | --- | --- | --- | --- | --- | --- | --- | --- |
| fit\_dfd\_log\_audit3 | 1 | 24 | 2142.663 | 2266.109 | -1047.331 |  | NA | NA |
| fit\_dfd\_log\_audit4 | 2 | 27 | 2146.360 | 2285.238 | -1046.180 | 1 vs 2 | 2.302 | 0.512 |

Moderation of Positive AOE on weekly drinking frequency

|  | Model | df | AIC | BIC | logLik | Test | L.Ratio | p-value |
| --- | --- | --- | --- | --- | --- | --- | --- | --- |
| fit\_dfd\_log\_ae\_positive3 | 1 | 24 | 2222.056 | 2345.710 | -1087.028 |  | NA | NA |
| fit\_dfd\_log\_ae\_positive4 | 2 | 27 | 2225.501 | 2364.612 | -1085.750 | 1 vs 2 | 2.555 | 0.465 |

Moderation of Negative AOE on weekly drinking frequency

|  | Model | df | AIC | BIC | logLik | Test | L.Ratio | p-value |
| --- | --- | --- | --- | --- | --- | --- | --- | --- |
| fit\_dfd\_log\_ae\_negative3 | 1 | 24 | 2221.028 | 2344.683 | -1086.514 |  | NA | NA |
| fit\_dfd\_log\_ae\_negative4 | 2 | 27 | 2222.264 | 2361.375 | -1084.132 | 1 vs 2 | 4.765 | 0.19 |

Moderation of AUDIT on largest drinking quantity in one day

|  | Model | df | AIC | BIC | logLik | Test | L.Ratio | p-value |
| --- | --- | --- | --- | --- | --- | --- | --- | --- |
| fit\_most\_drink\_log\_audit3 | 1 | 24 | 1680.786 | 1804.233 | -816.393 |  | NA | NA |
| fit\_most\_drink\_log\_audit4 | 2 | 27 | 1686.123 | 1825.001 | -816.062 | 1 vs 2 | 0.663 | 0.882 |

Moderation of Positive AOE on largest drinking quantity in one day


|  | Model | df | AIC | BIC | logLik | Test | L.Ratio | p-value |
| --- | --- | --- | --- | --- | --- | --- | --- | --- |
| fit\_most\_drink\_log\_ae\_positive3 | 1 | 24 | 1725.684 | 1849.338 | -838.842 |  | NA | NA |
| fit\_most\_drink\_log\_ae\_positive4 | 2 | 27 | 1728.317 | 1867.428 | -837.158 | 1 vs 2 | 3.367 | 0.338 |

Moderation of Negative AOE on largest drinking quantity in one day


|  | Model | df | AIC | BIC | logLik | Test | L.Ratio | p-value |
| --- | --- | --- | --- | --- | --- | --- | --- | --- |
| fit\_most\_drink\_log\_ae\_negative3 | 1 | 24 | 1735.242 | 1858.897 | -843.621 |  | NA | NA |
| fit\_most\_drink\_log\_ae\_negative4 | 2 | 27 | 1739.963 | 1879.075 | -842.982 | 1 vs 2 | 1.279 | 0.734 |

Moderation of AUDIT on alcohol-related consequences

|  | Model | df | AIC | BIC | logLik | Test | L.Ratio | p-value |
| --- | --- | --- | --- | --- | --- | --- | --- | --- |
| fit\_consq\_log\_audit3 | 1 | 24 | 1973.044 | 2096.491 | -962.522 |  | NA | NA |
| fit\_consq\_log\_audit4 | 2 | 27 | 1972.988 | 2111.866 | -959.494 | 1 vs 2 | 6.056 | 0.109 |

Moderation of Positive AOE on alcohol-related consequences

|  | Model | df | AIC | BIC | logLik | Test | L.Ratio | p-value |
| --- | --- | --- | --- | --- | --- | --- | --- | --- |
| fit\_consq\_log\_ae\_positive3 | 1 | 24 | 2123.832 | 2247.486 | -1037.916 |  | NA | NA |
| fit\_consq\_log\_ae\_positive4 | 2 | 27 | 2121.797 | 2260.909 | -1033.899 | 1 vs 2 | 8.034 | 0.045 |

Condition x Time interaction effects on alcohol-related consequences among high positive AOE


|  | Value | Std.Error | DF | t-value | p-value | cohen\_d | LCL\_d | UCL\_d |
| --- | --- | --- | --- | --- | --- | --- | --- | --- |
| (Intercept) | 0.808 | 0.053 | 340 | 15.128 | 0.000 | 1.328 | 1.156 | 1.501 |
| conditionWaitlist | 0.066 | 0.074 | 249 | 0.890 | 0.374 | 0.108 | -0.130 | 0.347 |
| timeone\_month | -0.277 | 0.061 | 340 | -4.565 | 0.000 | -0.455 | -0.651 | -0.260 |
| timetwo\_months | -0.404 | 0.069 | 340 | -5.885 | 0.000 | -0.665 | -0.886 | -0.443 |
| timesix\_months | -0.272 | 0.078 | 340 | -3.497 | 0.001 | -0.447 | -0.698 | -0.197 |
| conditionWaitlist:timeone\_month | -0.085 | 0.085 | 340 | -0.990 | 0.323 | -0.139 | -0.415 | 0.136 |
| conditionWaitlist:timetwo\_months | -0.035 | 0.096 | 340 | -0.366 | 0.715 | -0.058 | -0.367 | 0.251 |
| conditionWaitlist:timesix\_months | -0.173 | 0.113 | 340 | -1.536 | 0.125 | -0.285 | -0.648 | 0.078 |

Condition x Time interaction effects on alcohol-related consequences among low positive AOE


|  | Value | Std.Error | DF | t-value | p-value | cohen\_d | LCL\_d | UCL\_d |
| --- | --- | --- | --- | --- | --- | --- | --- | --- |
| (Intercept) | 1.016 | 0.055 | 379 | 18.604 | 0.000 | 1.537 | 1.375 | 1.699 |
| conditionWaitlist | -0.149 | 0.077 | 293 | -1.945 | 0.053 | -0.226 | -0.454 | 0.002 |
| timeone\_month | -0.388 | 0.071 | 379 | -5.479 | 0.000 | -0.586 | -0.796 | -0.377 |
| timetwo\_months | -0.404 | 0.074 | 379 | -5.464 | 0.000 | -0.612 | -0.831 | -0.392 |
| timesix\_months | -0.446 | 0.072 | 379 | -6.189 | 0.000 | -0.675 | -0.889 | -0.461 |
| conditionWaitlist:timeone\_month | -0.017 | 0.103 | 379 | -0.167 | 0.868 | -0.026 | -0.333 | 0.281 |
| conditionWaitlist:timetwo\_months | 0.042 | 0.110 | 379 | 0.383 | 0.702 | 0.064 | -0.262 | 0.390 |
| conditionWaitlist:timesix\_months | 0.100 | 0.107 | 379 | 0.930 | 0.353 | 0.151 | -0.167 | 0.470 |

Moderation of Negative AOE on alcohol-related consequences

|  | Model | df | AIC | BIC | logLik | Test | L.Ratio | p-value |
| --- | --- | --- | --- | --- | --- | --- | --- | --- |
| fit\_consq\_log\_ae\_negative3 | 1 | 24 | 2078.878 | 2202.532 | -1015.439 |  | NA | NA |
| fit\_consq\_log\_ae\_negative4 | 2 | 27 | 2084.322 | 2223.434 | -1015.161 | 1 vs 2 | 0.556 | 0.907 |
